# Exploring Students’ Use of a Mobile Application to Support Their Self-Regulated Learning Processes

**DOI:** 10.3389/fpsyg.2022.793002

**Published:** 2022-03-14

**Authors:** Martine Baars, Sanyogita Khare, Léonie Ridderstap

**Affiliations:** ^1^Department of Psychology, Education, and Child Studies, Erasmus School of Social and Behavioral Science, Rotterdam, Netherlands; ^2^Faculty Office, Erasmus School of History, Culture, and Communication, Rotterdam, Netherlands

**Keywords:** self-regulated learning, mobile application, motivation, self-efficacy, monitoring

## Abstract

Being able to self-regulate one’s learning is essential for academic success but is also very difficult for students. Especially first year students can be overwhelmed with the high study load and autonomy in higher education. To face this challenge, students’ monitoring and self-regulated learning (SRL) processes are crucial. Yet, often students are not aware of effective SRL strategies or how to use them. In this study, the use of a mobile application with gamification elements (i.e., Ace Your Self-Study App) to support first-year university students’ SRL processes was investigated. In Study 1a, the Ace your self-study app was implemented in a first-year psychology course, and students’ SRL skills, motivation, self-efficacy, app use and satisfaction, and performance were measured. The results showed a significant increase in autonomous motivation, controlled motivation, and metacognitive self-regulation skills (MSR-R) across the 5-week course. Moreover, students who used the mobile application with gamified elements showed higher autonomous motivation. Nevertheless, most students used the app only for a limited number of self-study sessions. In Study 1b, students’ self-study experiences were captured using focus group interviews to shed some more light on why students did or did not use the app. The results show that if students feel they do not need support for their SRL processes during self-study, they are less inclined to use the app. Specifically, regarding using study strategies, it was found that only if students’ strategies do not work well in their perception, they feel the need to change their way of studying and choose another strategy. These results are discussed in the context of theory on SRL and how to support it.

## Introduction

First year students starting in higher education can be overwhelmed by the course load they encounter and the challenges this poses to their study skills. To self-regulate their study process, students need to be able to accurately keep track of their own learning process (i.e., monitoring) and use that information to regulate their learning process (e.g., Zimmerman, 2002, 2008). Yet, research has shown that students are often not capable of self-regulating their learning processes. That is, without instructional guidance, they find it difficult to accurately judge their own learning processes (e.g., Dunning et al., 2003; Dunlosky and Lipko, 2007) and consequently, regulation of the learning processes is hampered (e.g., Pressley, 1995; Dunlosky and Rawson, 2012). This problematic cycle of suboptimal *self-regulated learning* (SRL) could stand in the way of academic success and the goal to become life-long learners. Especially because students often do not get instruction about how to study and are largely unaware of *learning strategies* that could help them to study effectively (e.g., McCabe, 2011; Bjork et al., 2013; Dirkx et al., 2019). Therefore, the main aim of this study is to investigate the use of a mobile application with *gamification* elements to support SRL processes of first year students in higher education.

### Self-Regulated Learning and How to Support it

Self-regulated learning can be defined as the degree to which people are “metacognitively, and behaviorally active participants in their own learning process” (Zimmerman, 1989, p. 4). Zimmerman (2008) describes a cyclical model of SRL which entails three phases: the forethought, performance, and reflection phase. First, students start the cycle with the forethought phase during which they can prepare their study session by, for example, setting a goal for the session or analyzing the task for that session. After the forethought phase, the performance phase follows. During this phase, students use strategies to process the learning materials (e.g., summarizing or self-explaining) and keep track of their learning processes (i.e., self-monitoring). Finally, in the reflection phase, students evaluate their study session, for example by making self-judgments about their learning and satisfaction.

Research has shown that metacognitive processes, such as monitoring and control, which allow students to self-regulate or self-manage their learning processes and choose which cognitive strategies to use, are crucial for academic success (e.g., Thiede et al., 2003; Broadbent and Poon, 2015; Dent and Koenka, 2015). These findings align with the model of SRL by Zimmerman (2002, 2008) as both cognitive and metacognitive processes are crucial in going through the three phases of SRL. Metacognitive processes for example are, students setting learning goals, self-monitoring learning processes, and regulating or managing their learning processes. Using study strategies, for example during the performance phase, entails all kinds of cognitive processes, such as summarizing, elaboration, or self-testing but also management strategies such as time management.

Yet, very often students are not aware of metacognitive or cognitive strategies that they can use to regulate their own learning (McCabe, 2011; Bjork et al., 2013; Dirkx et al., 2019). Moreover, research has shown that if students do not get instructional support on how to monitor their learning processes, their insight in their own learning process and how to proceed is generally very poor. Specifically, students were found to overestimate their understanding of learning materials (such as texts, e.g., Thiede et al., 2009) and their memory of learning materials (such as word pairs, e.g., Dunlosky and Lipko, 2007) when no additional instructional support was provided. Inaccurate self-monitoring can have detrimental effects on the learning process. For example, in a study by Dunlosky and Rawson (2012), retention of the learning materials was lower because of premature termination of study by students who overestimated their performance.

Importantly, providing instructional support to help students self-regulate their learning has shown to be beneficial in terms of SRL processes (e.g., Devolder et al., 2012), strategy use, and learning outcomes (e.g., Dignath and Büttner, 2008). Interventions to support SRL processes based on metacognitive theories, like metacognitive reflection (Dignath and Büttner, 2008) and planning strategies (Dignath et al., 2008), were found to improve strategy use and learning outcomes. In a review by Devolder et al. (2012) on supporting SRL in computer-based learning environments, it is concluded that SRL scaffolds support SRL processes of the learners. Yet, very often studies only prompted cognitive strategies (e.g., self-explaining) and did not prompt aspects from the other phases of SRL. Another review on supporting SRL in online learning environments concluded that prompting SRL processes such as planning benefitted students SRL behaviors and performance (Wong et al., 2019). The authors also noted that many studies only prompted and measured behaviors related to one of the SRL phases and that it might be better to prompt and measure aspects from multiple SRL phases.

In addition, more recent work stresses the importance of combining cognitive and metacognitive prompts to support SRL (Nückles et al., 2020). Prompting cognitive (e.g., summarizing and note taking) and metacognitive (e.g., planning, monitoring, and reflection) strategies were found to be most effective to support SRL. Moreover, research has shown that the most optimal sequence of prompts consists of metacognitive prompts first followed by cognitive prompts (Roelle et al., 2017a).

Next to prompting students to use cognitive and metacognitive strategies, student’s self-efficacy plays an important role in SRL (e.g., Panadero et al., 2017). That is, if students’ self-efficacy beliefs about their capabilities are low they are likely to avoid tasks compared to students who have high self-efficacy beliefs which make it likely they will participate in tasks (e.g., Schunk, 1990). This could mean that students with low self-efficacy will not use the cognitive or metacognitive strategies that are prompted as much as the students who have higher self-efficacy beliefs. On the other hand, students with higher self-efficacy and who use more learning strategies were found to have higher task performance. In turn, higher performance was linked to higher self-efficacy on subsequent learning tasks (Wilson and Narayan, 2016). Yet, engaging in SRL could provide students with a deeper understanding of the learning task, which could enable them to perform better and experience success. This could result in increased feelings of competence which in turn positively affect self-efficacy beliefs (e.g., Schunk, 1996; Paris and Paris, 2001).

In sum, it seems promising to support students’ SRL processes by designing effective instructional support in which both metacognitive and cognitive strategies are elicited and students are stimulated to go through all the SRL phases accordingly.

### Mobile Technology to Support SRL

To make sure that these instructional prompts will be provided just in time during the learning processes (see Van Merriënboer et al., 2002), mobile technology seems promising to support SRL. That is, almost every student has a mobile phone and with this mobile device SRL support can be brought close to the student’s learning process at anytime and anywhere. Yet, there has been very little research about the use of mobile devices to support SRL processes. In a study by Tabuenca et al. (2015) graduate students used a mobile device to track time during their learning processes. The results of their study showed that tracking time during the learning process had a positive effect on time management. These results suggest that using mobile devices to support SRL processes such as time management are very promising (Tabuenca et al., 2015). Similarly, a recent study by Broadbent et al. (2020) found that combining an online SRL training module with a mobile application to capture daily diaries on study activities and affect, had positive effects in terms of resource management (i.e., time and space), and metacognitive and cognitive strategies. This study has shown that a domain-independent intervention was successful in improving students’ SRL strategies. Interestingly, when students only used the mobile-app for daily diaries on their study activities, they did not seem to improve their SRL strategies compared to a control condition. The authors highlight that only self-monitoring *via* a daily diary is probably not enough if someone does not know *how* to self-regulate his or her learning. Hence, the online training on SRL containing information on the three SRL phases combined with prompts *via* the mobile application at the beginning and ending of a study session seem to really support students to self-regulate their learning.

In addition to the few studies on using mobile technology to support SRL, there is abundant literature on supporting SRL in computer-supported or online environments. For example, in a review by Wong et al. (2019), it is reported that 14 out of the 35 studies reviewed used prompts as a means to support SRL. Another six studies out of 35 used a combination of prompts and feedback to support SRL. Also, 10 studies used integrated systems in the learning environment to support SRL. Other approaches that were reported in this review study were self-monitoring form (one study), e-learning (one study), training (two studies), or conceptualization of learning outcomes (one study). Hence, this review shows that although there is a variety of SRL support used in online learning environments, prompting or a combination of prompting with for example feedback is a well-researched way of supporting SRL.

Next to using mobile technology to provide instructional support for SRL, it can also provide the students with gamification elements to boost their motivation and SRL performance. Gamification can be defined as adding game elements to a non-game context (Buckley et al., 2018). Levels, points, and scoreboards are examples of gamification elements that can increase students’ motivation and performance. Specifically, these gamification elements can provide students with clear goals and rewards, which in turn keeps them engaged and motivated to use the materials offered (e.g., Su and Cheng, 2015; Mekler et al., 2017).

**Figure 1:**
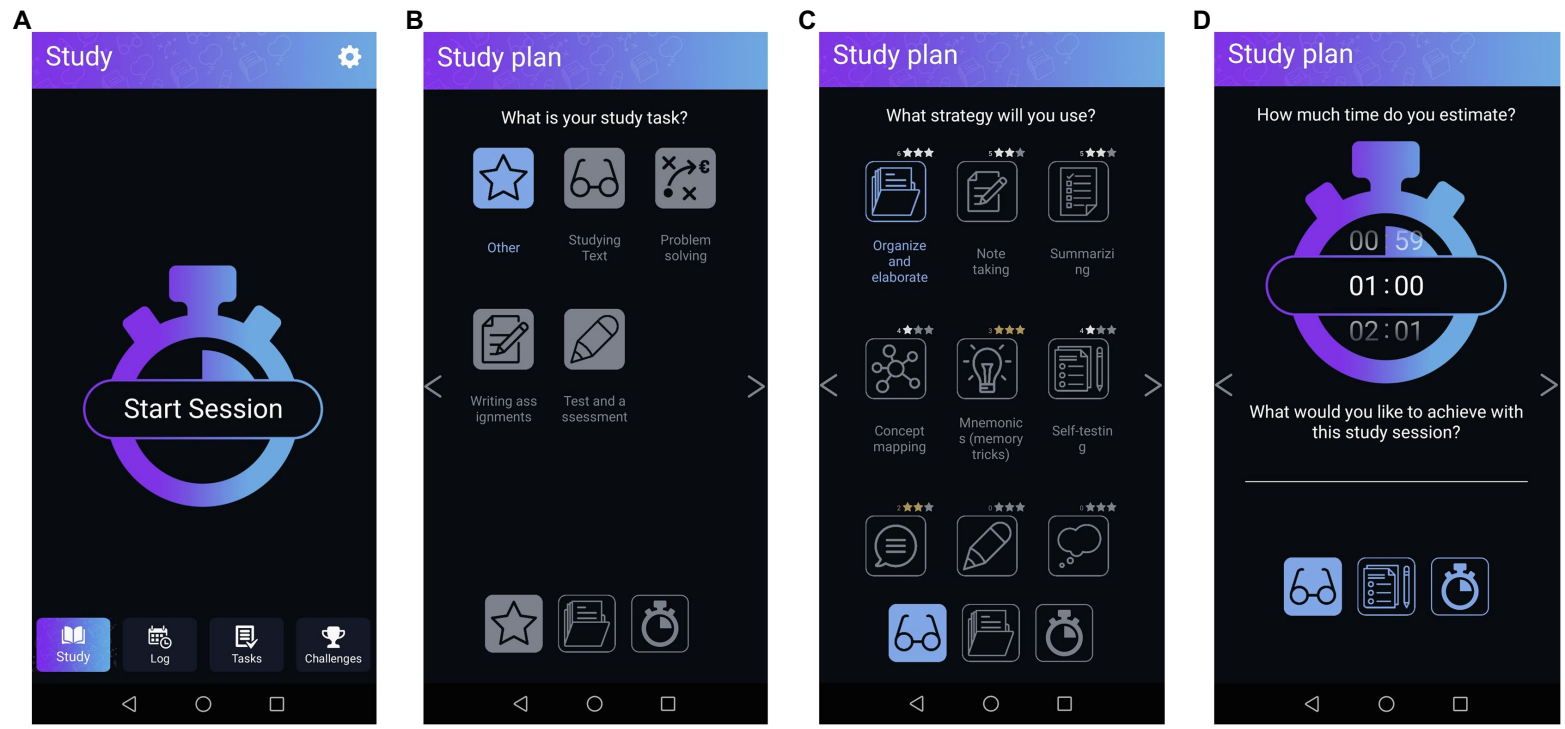
Screenshots from the forethought phase. **(A)** shows the first “study plan” screen to sta1t a session. **(B)** Shows the second “study plan” screen at which students choose the type of task. **(C)** Shows the third “study plan” screen at which students choose a strategy. **(D)** Shows the fourth “study plan” screen at which students can set the time and fill out their goal [adapted from Baars et al., submitted (see footnote 1)].

**Figure 2:**
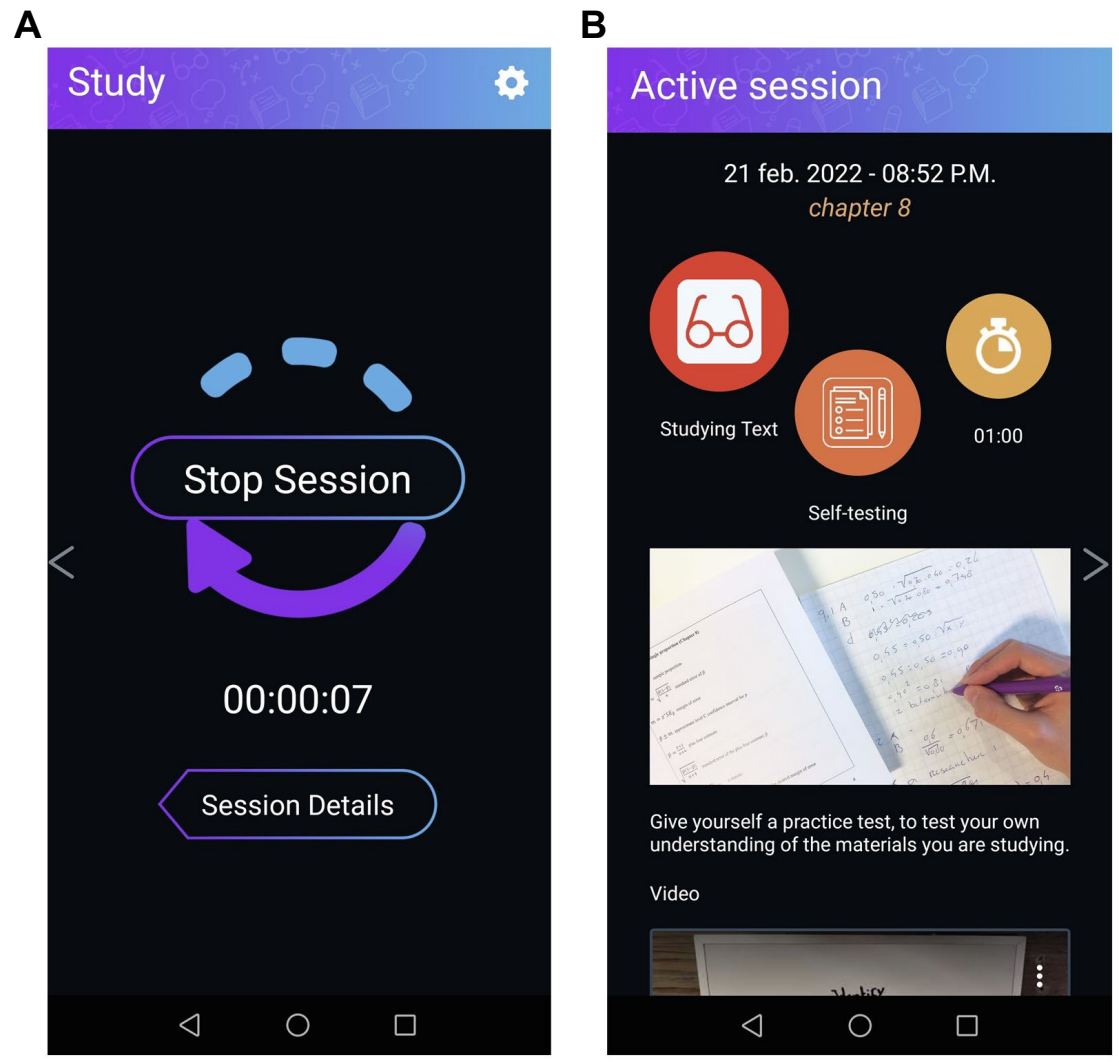
Screenshots from the performance phase. **(A)** Shows the defaults screen during the performance phase, which shows a timer. **(B)** Shows the summary of the “study plan” made in the forethought phase (adapted from see footnote 1).

**Figure 3:**
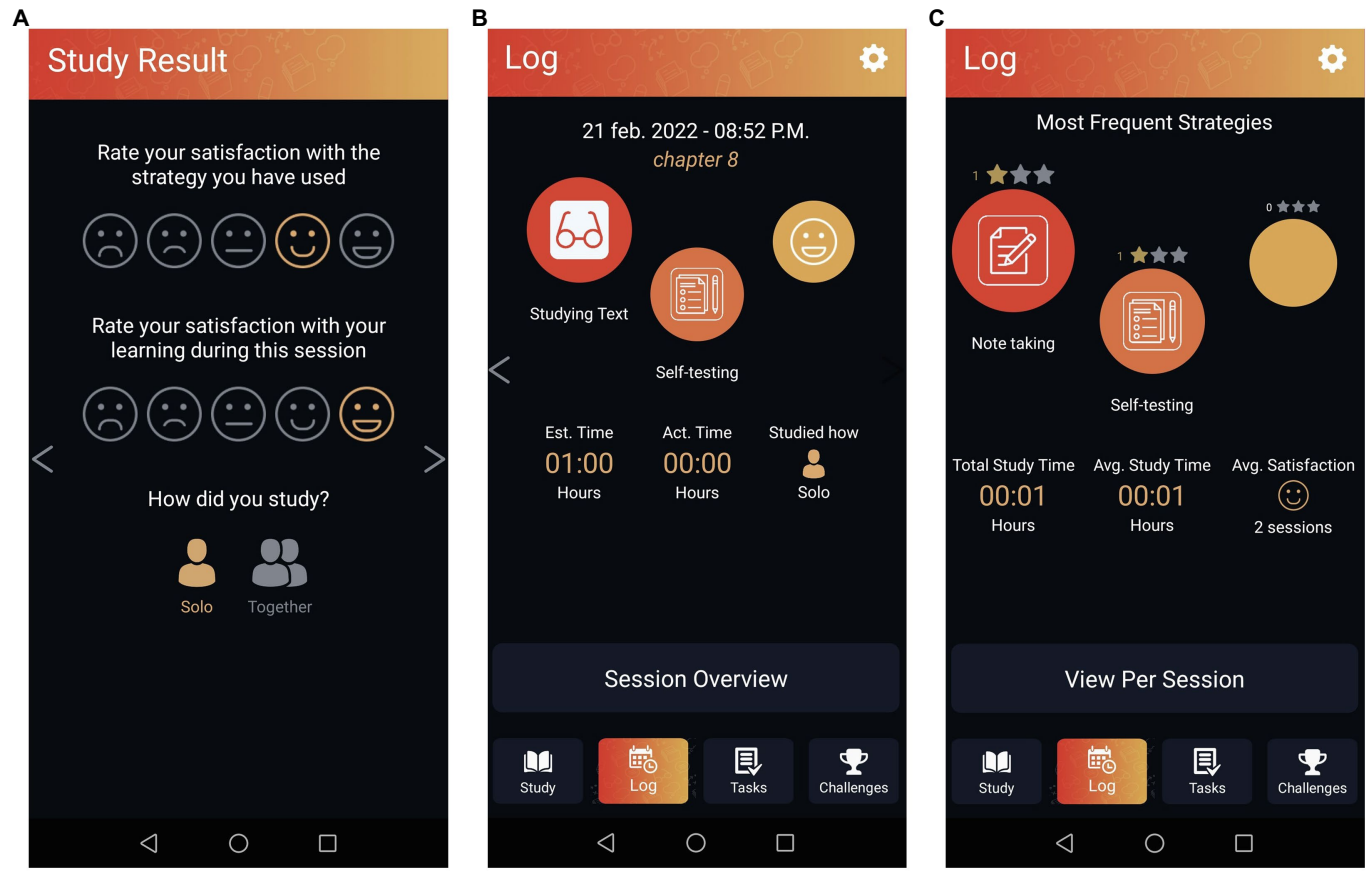
Screenshots from the reflection phase. **(A)** Shows the two ratings that students have to fill out. **(B)** Shows the log for a single session. **(C)** Shows the log across sessions (adapted from see footnote 1).

Building upon the model of SRL (Zimmerman, 2002, 2008) and extending earlier studies on using mobile applications or computer-supported applications to support SRL, a mobile application to support SRL strategies during self-study was developed (see Baars et al., submitted)^1^: the Ace Your Self-study App (Figures 1–3, download *via* App store or Play store). The App was designed to help users go through three phases of SRL: forethought, performance, and evaluation. To support the forethought phase, in the App, students are prompted to start a study session and create a study plan by selecting the type of task; a suitable study strategy, deciding how much time they will need for the session, and filling out a goal of their study session (see Figure 1).

In the app, users can choose evidence-based cognitive strategies (e.g., note taking, summarizing, and concept mapping) and receive an explanation on how to use them (see Appendix A). This way they can enter the performance phase well-prepared. Moreover, if during the performance phase students need to look at their study plan, the app provides a brief overview of their choices (see Figure 2).

Once students decide to stop their current session, they are prompted to reflect on their learning process. They rate their satisfaction with learning in general and with the strategy they have used. Also, students select whether they worked alone or together with other students. Then, a log appears providing a summary of a single session or across sessions (see Figure 3).

**Figure 4:**
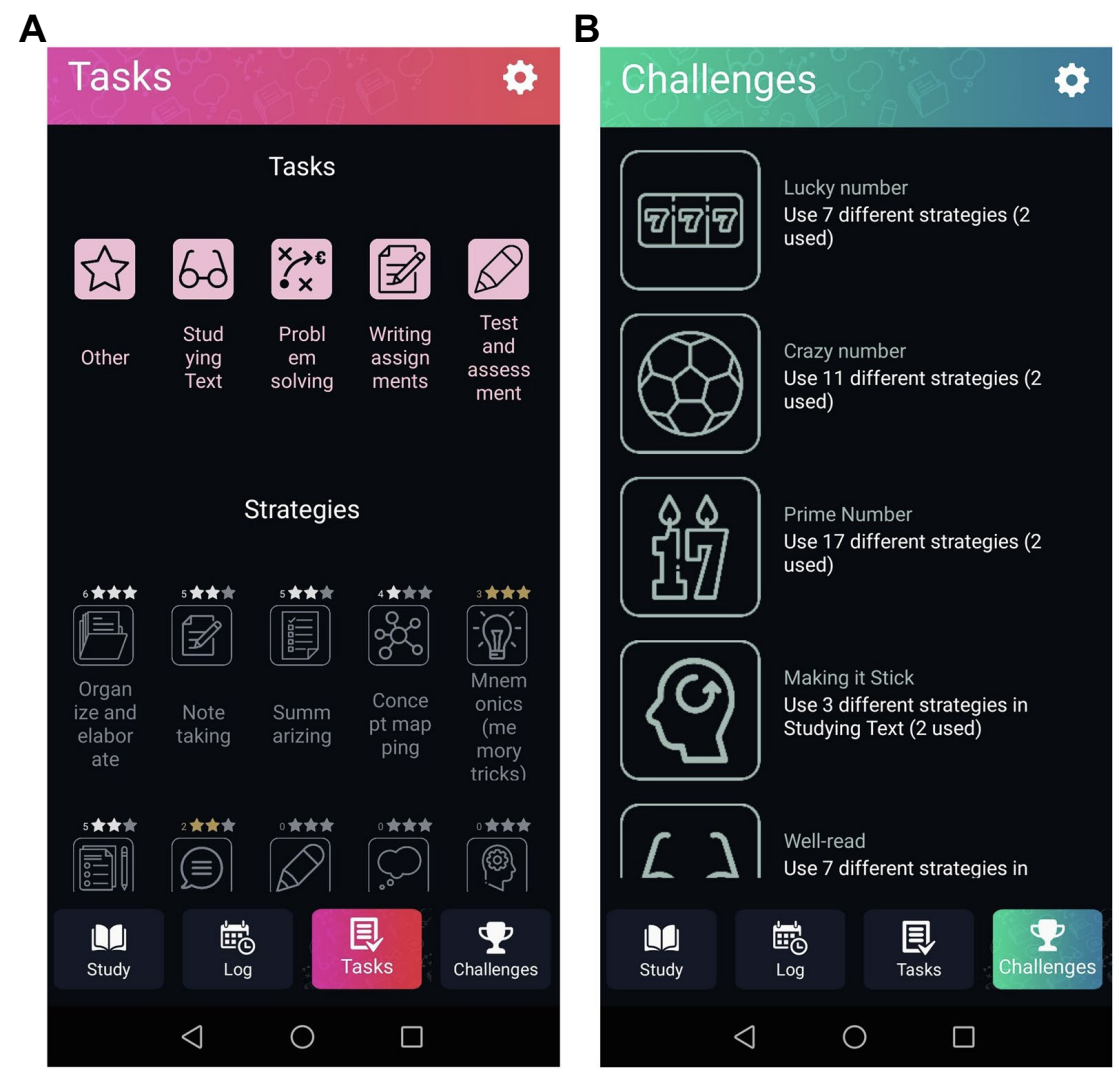
Screenshots of gamifzcation elements in app. **(A)** Shows the overview of the strategies with the level of use depicted in stars. **(B)** Shows the challenges students can take when using the app (adapted from see footnote 1).

In the Ace your self-study app, 22 cognitive study strategies are offered. As research has shown, students are often not aware of the different types of study strategies they can use (McCabe, 2011; Bjork et al., 2013; Dirkx et al., 2019). Therefore, gamification elements were added to the app to stimulate students to use a variety of study strategies during their self-study. In the tab “Tasks” in the app, students can find all the types of tasks and the strategies that can be used for those tasks (Figure 4). Stars were added to each strategy to create levels in using the study strategies from the app. The tab “Challenges” provides the student with some challenges in terms of using a variety of learning strategies. For example, “Lucky number, use seven different strategies” (see Appendix B).

### Motivation to Self-Regulate Learning

According to the self-determination theory (SDT; Deci and Ryan, 2000; Ryan and Deci, 2000a,b, 2020; Pelletier et al., 2001), intrinsic motivation and the internalisation of originally extrinsic behaviors can be enhanced if the basic psychological needs are satisfied. The three psychological needs are the need for autonomy, relatedness, and competence. The need for autonomy refers to the need to feel a sense of initiative and ownership in one’s actions. Autonomy can be supported by experiences of interest and value, but it can be undermined by being externally controlled (by punishment or rewards). The need for relatedness refers to the need to feel connected to others and have a sense of belonging. Relatedness is supported by conveying respect and caring. The need for competence refers to the feeling of mastery, and being able to grow. Competence can be supported in well-structured environments that allow for opportunities for growth, positive feedback, and challenges (Ryan and Deci, 2020).

In the SDT of motivation the quality of motivation which is determined by the reasons driving students behavior, is considered more important than the total amount of motivation when predicting psychological health and well-being, effective performance, and conceptual and deep learning (e.g., Vansteenkiste et al., 2006; Deci and Ryan, 2008). There is an important distinction between self-determined and controlled motivation. Students would engage in self-determined motivated actions if they would do this freely and volitionally. In contrast, students would engage in controlled motivated actions because of interpersonal or intrapsychic force. If student behavior is self-determined, regulation of learning would be based on choice, whereas if student behavior is controlled, regulation of learning would be based on compliance (Deci et al., 1991).

Hence, motivation of students can be expressed in terms of autonomy (Ryan and Deci, 2000a,b, 2020). Deci and Ryan (2000) proposed a self-determined continuum ranging from amotivation to intrinsic motivation. On this continuum of the degree of experienced autonomy, there are several types of motivation that can be conceptualized. Students with a high degree of autonomous motivation experience volition and psychological freedom. They study because it brings them satisfaction or because the subject is interesting to them (i.e., intrinsic motivation). Studying could also be valuable for development or attaining personal goals (i.e., identified motivation). More to the other side of the continuum of the degree of experienced autonomy, students who score high on controlled motivation experience a low degree of autonomy and experience pressure. This pressure can come from within the student (i.e., introjected motivation) when, for example, students feel pressure to avoid feelings of shame. This pressure can also come from an external source, such as demands from a parent or teacher (i.e., external motivation). It is important to note here that research has shown that intrinsic motives can coexist with extrinsic motives (e.g., Covington and Müeller, 2001). Moreover, these different types of motivation can operate at different levels (Vallerand, 1997), such as trait, contextual (e.g., school level), and the situational level (e.g., for a specific subject or moment). In the current study, we investigated specific motivation for self-studying the learning materials of the course. The type of motivation on the continuum of experienced autonomy students possess is relevant in terms of persistence, well-being, and learning outcomes. Specifically, more autonomously motivated students were found to have better text comprehension (e.g., Vansteenkiste et al., 2004) and (self-reported) academic achievement (e.g., Vansteenkiste et al., 2009; Taylor et al., 2014). Furthermore, autonomous motivation in terms of having interest for a subject, has been associated with better problem-solving performance (for a review, see Mayer, 1998) and better SRL abilities such as effort regulation (i.e., controlling effort and attention) and metacognitive strategy use (i.e., checking and correcting one’s own learning behavior; Vansteenkiste et al., 2009; León et al., 2015). The relation between autonomous motivation and SRL skills was also shown in work by Pintrich (1999) who found that students who indicated higher levels of interest for a course (i.e., an autonomous reason for studying), were more likely to use strategies to monitor and regulate their learning. More recently, Baars and Wijnia (2018) and Wijnia and Baars (2021) have shown that secondary education students who were more autonomously motivated improved more in their monitoring skills after a SRL training.

To conclude, the role of motivation in using SRL skills cannot be underestimated. It is likely that students who would be more autonomously motivated will engage in SRL behaviors more often in general but will probably also be more inclined to make use of SRL supports such as the Study app.

### The Current Study

The current study consisted of two parts, Study 1a and Study 1b. First, in Study 1a, SRL activities by first year students as measured in the Ace your self-study app were investigated in relation to SRL skills, motivation, self-efficacy, satisfaction (with study strategy and learning), and performance across a 5-week course. Moreover, the effect of using the study app with or without gamification elements on students’ app usage (i.e., frequency and duration) and students’ motivation (autonomous and controlled), self-efficacy, satisfaction (with study strategy and learning), SRL skills, and course performance was investigated. In study 1b, using a qualitative approach, students’ experiences using the app during Study 1a were investigated *via* focus groups interviews.

## Study 1a: Using the Ace Your Self-Study App

In this study, the usage of the Ace Your Self-study app, from here on called the Study app, was investigated by the following research questions. The first research question is:

What is the relation between Study app usage (i.e., frequency and duration) and students’ motivation (autonomous and controlled), self-efficacy, satisfaction (with strategy and learning), SRL skills, and course performance?

As SRL and motivation are related to each other (Pintrich, 1999; Baars and Wijnia, 2018; Wijnia and Baars, 2021), we have measured the SRL activities in the app as indication of students’ engagement in SRL. We expect the frequency (i.e., number of sessions) and the duration of using the Study app (i.e., total time) will be positively related to pretest autonomous motivation (H1.1), pretest self-efficacy (H 1.2), satisfaction ratings of study strategy and learning in the app (H1.3), pretest SRL skills (H1.4), and course performance (H1.5).

In addition, the effect of the Study app with or without gamification elements (i.e., levels and challenges) was investigated by randomly assigning half of the students to a second version of the Study app in which gamification elements are added to the original version. Therefore, the second research question is: What is the effect of gamification elements in the Study app on students’ app usage (i.e., frequency and duration) and students’ motivation (autonomous and controlled), self-efficacy, satisfaction (with study strategy and learning), SRL skills across the course (from pre- to posttest), and course performance?

We expect that students in the Study app with gamification elements (Study game app) condition will show higher app usage (both duration and frequency; H2.1), higher autonomous motivation (H2.2), higher satisfaction ratings of study strategy and learning in the app (H2.3), higher self-efficacy, more SRL skills (H2.4), and higher performance (H2.5) compared to students in the non-gamified (Study app) condition.

### Method

We created an Open Science Framework (OSF) page for this project, where all materials, and a detailed description of the procedure are provided (DOI 10.17605/OSF.IO/98NYH). Data are available upon request.

#### Participants and Procedure

We offered the Study app in the first-year practical on Problem-Based Learning (PBL) and study skills. Out of 912 students who were enrolled in the practical, 505 students downloaded the Ace your Self-Study app. If students could not or would not use the mobile application, they were invited to use a document containing the same content information as available in the mobile application, which was provided as an appendix to their practical guide. One hundred and ninety first year students (*M_age_* = 20.22, *SD* = 4.05, 139 females, 49 males, and two other) filled out both the pre and post survey. From this sample, 99 students (*M_age_* = 20.43, *SD* = 4.58, 70 females, 28 males, and one other) used the app, which allowed us to retrieve their backend data logged by the mobile application showing their activities using the app. From the sample with both completed surveys and app data, 52 (*M_age_* = 19.98, *SD* = 2.92, 35 females, and 17 males) participants had been randomly assigned to the Study app and 47 (*M_age_* = 20.85, *SD* = 5.67, 35 females, 11 males, and one other) to the Study Game app.

During the first practical meeting (small-group, tutorial meeting), students were invited to take part in this study. Using a Qualtrics survey,^2^ students were provided with information on the current study and asked for their consent regarding using their data from the survey, the practical (i.e., performance), and the Study app for the purposes of the current study only. If students choose to participate in the study, they were presented with a survey about their motivation, self-efficacy, and SRL skills (i.e., pretest). During the practical, students received a homework assignment in meeting 1 (week 1) in which they were instructed to use the Study app for their self-study sessions during the whole course of the practical (Appendix C). In week 4 of the practical, students took part in a reflection exercise during a meeting to reflect on and evaluate their study behaviors using the Study app (Appendix C). This exercise was guided by their tutor. At the end of the practical during the last meeting, a survey on motivation, self-efficacy, SRL skills, and satisfaction with the Study app features was administered (i.e., posttest).

#### Materials and Measurements

##### SRL Skills

Two scales of the MSLQ, the metacognitive self-regulation (MSR) and the time and study environment (TSE) scales were filled in by the participants *via* a Qualtrics questionnaire. The MSLQ consists of 15 subscales with 81 items, which measure motivation, learning strategies, and management of resources with a Likert scale from 1 (*not at all true for me*) to 7 (*very true of me*; Pintrich, 1991, 2004). For this study, we used the adjusted scale “MSR revised” (i.e., MSR-R; Tock and Moxley, 2017) which comprises of nine items with an average weighted reliability of 0.78, and the “time and study environment scale” (i.e., TSE) from the MSLQ which comprises of eight items with a Cronbach’s alpha of 0.76 (Pintrich, 1991). The scales were scored by taking the average of the items per scale. In this study, the MSR-R demonstrated a low reliability of 0.57 in the pretest and 0.64 in the posttest. Similarly, the TSE showed a reliability of 0.68 in the pretest and 0.66 in the posttest.

##### Motivation

Students filled out a 16-item task-specific version of the academic self-regulation scale (Vansteenkiste et al., 2004), for which students had to indicate why they engaged in self-studying the learning materials in the course (i.e., “I engaged in self-study for this course because…”). The scale consisted of four subscales: external (e.g., “… because I am supposed to do so”), introjected (e.g., “… because I would feel guilty if I did not do it”), identified (e.g., “… because I could learn something from it”), and intrinsic motivation (e.g., “… because I found it interesting”). Items were measured on a five-point Likert-type scale ranging from 1 (not at all true) to 5 (totally true). The scales were scored by taking the average of the items per scale. The four subscales were combined into an autonomous motivation composite (intrinsic and identified motivation) and a controlled motivation composite (introjected and external motivation; *cf.* Vansteenkiste et al., 2004). Both composite scales showed good reliability in the pretest (0.85 for autonomous and 0.83 for controlled motivation) as well as in the posttest (0.88 for autonomous and 0.84 for controlled motivation).

##### Self-Efficacy

Students were asked to indicate their degree of confidence in their ability to be successful in self-studying the learning materials offered in this course by recording a number from 0 to 100 (Bandura, 2006).

##### Learning Performance

Learning performance was measured by collecting the exam grades for the practicum.

##### Ace Your Self-Study App Log Data

From the Ace your self-study app, log data were collected per session that was initiated using the app, on the type of task, strategy choice, estimated and elapsed time, goals, and satisfaction ratings about learning and about the strategy that was used. For the purpose of this study, for each participant, the number of sessions and the duration of those sessions during the 5-week course were calculated. We defined a study session using the mobile application as any session that lasted between 1 min and 12 h. Sessions that were shorter or longer were discarded from the analyses for the current study.

##### Evaluation of User-Friendliness Study App

In the posttest students received seven items on navigating the app, content of the app, errors, bias, and whether one would recommend the app to others, in order to evaluate the user-friendliness of the app (Appendix D).

### Results

Descriptive data of study app usage (frequency and duration), study app ratings (average strategy rating and satisfaction with learning), pre-and posttest measures (motivation, self-efficacy, and SRL skills), and course grades are displayed in Table 1.

**Table 1:** Means and SDs for Study App usage, Study App rating, motivation, self-efficacy, self-regulated learning (SRL) skills variables, and final practical grade.

Variable (Range)	Overall	Gamified	Standard
*Study App Usage*			
Number of sessions	4.11 (4.07)	4.06 (3.79)	4.17 (4.40)
Session Duration in Minutes	112.58 (80.52)	108.52 (80.94)	117.08 (80.69)
*Study App Rating*			
Strategy Rating (1–5)	3.94 (0.80)	3.98 (0.70)	3.89 (0.90)
Satisfaction with Learning (1–5)	3.69 (0.71)	3.73 (0.65)	3.65 (0.77)
*Motivation*			
Pre-test Autonomous Motivation (1–5)	4.03 (0.59)	4.12 (0.55)	3.93 (0.62)
Post-test Autonomous Motivation (1–5)	4.15 (0.58)	4.30 (0.54)	3.98 (0.58)
Pre-test Controlled Motivation (1–5)	2.18 (0.73)	2.24 (0.74)	2.11 (0.71)
Post-test Controlled Motivation (1–5)	2.28 (0.77)	2.35 (0.80)	2.21 (0.72)
*Self-efficacy*			
Pre-test Self-efficacy (0–100)	72.20 (13.14)	74.12 (12.43)	70.09 (13.70)
Post-test Self-efficacy (0–100)	72.01 (13.24)	74.60 (13.17)	69.15 (12.86)
*SRL Skills*			
Pre-test MSR-R (1–5)	3.55 (0.47)	3.61 (0.47)	3.48 (0.45)
Post-test MSR-R (1–5)	3.65 (0.46)	3.67 (0.48)	3.63 (0.45)
Pre-test TSE (1–5)	4.05 (0.47)	4.11 (0.47)	3.98 (0.47)
Post-test TSE (1–5)	3.98 (0.49)	4.05 (0.47)	3.89 (0.51)
*Final Course Grade*			
Grade (0–100)	63.16 (13.48)	65.20 (12.67)	60.91 (14.13)

**Figure 5:**
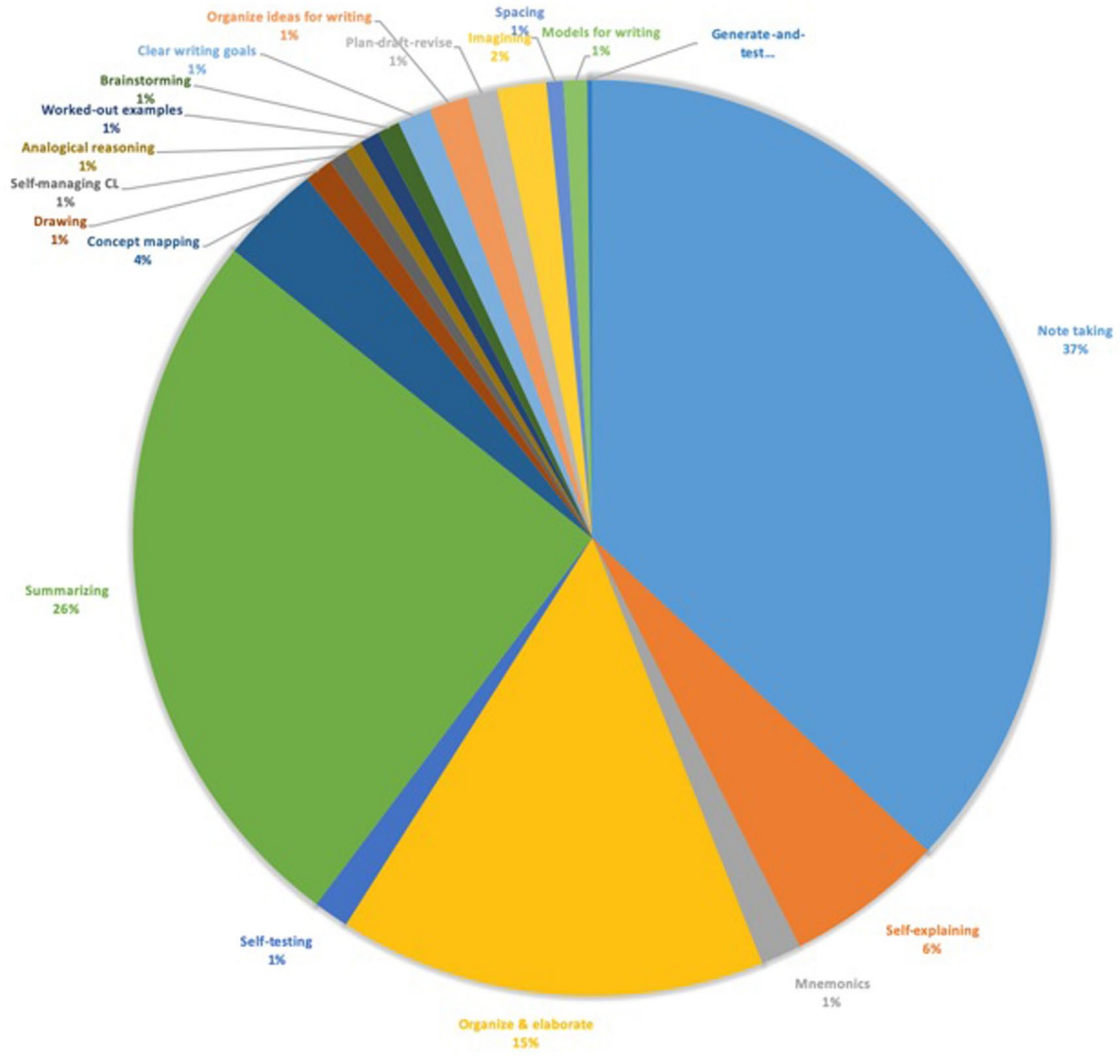
Percentage of learning strategies used in study app.

Figure 5 provides an overview of the percentage of sessions during which one of the types of learning strategies was used by the participants in the study app. The most commonly used strategy was note taking (37%), followed by summarizing (26%), organize and elaborate (15%), and self-explaining (6%). The other strategies only made up 2 or 1% of the total of strategies that were chosen during the study sessions registered by the app. These data show the limited number of strategies students were using in the app during this study.

In order to check for random assignment to the standard and gamified conditions, pretest motivation (autonomous and controlled), self-efficacy, and SRL skills (MSR-R and TSE) were compared between the two conditions. An independent-samples *t* test revealed no significant differences between the conditions in pre-survey autonomous motivation, *t*(97) = −1.59, *p* = 0.116, controlled motivation, *t*(97) = −0.93, *p* = 0.354, self-efficacy, *t*(97) = −1.53, *p* = 0.128, MSR-R scores, *t*(97) = −1.39, *p* = 0.169, and TSE scores, *t*(97) = −1.37, *p* = 0.174.

#### Correlations: Pretest Variables, App Usage, and App Ratings, and Course Performance

Table 2 displays Pearson’s correlations between the pretest variables (motivation, self-efficacy, and SRL skills), Study app usage variables (number of sessions and session duration), Study app rating variables (strategy rating and satisfaction with learning), and course performance (exam grade). Two-tailed tests of significance were conducted. At the 0.01 level, the following pairs of variables were significantly positively correlated: average strategy rating and satisfaction with learning (*r* = 0.698), TSE scores and satisfaction with learning (*r* = 0.358), TSE and MSR-R scores (*r* = 0.478), MSR-R scores and autonomous motivation (*r* = 0.419), and autonomous motivation and TSE scores (*r* = 0.404). At the 0.05 level, the following pairs of variables were significantly positively correlated: TSE scores and strategy rating (*r* = 0.230), autonomous motivation and satisfaction with learning (*r* = 0.236), autonomous motivation and self-efficacy (*r* = 0.254) MSR-R scores and self-efficacy (*r* = 0.211), and TSE scores and exam grade (*r* = 0.212). Furthermore, autonomous motivation and average session duration were significantly negatively correlated at the 0.01 level (*r* = −0.251).

**Table 2:** Pearson’s correlations between pre-test variables, Study App usage variables, and Study App rating variables and grades.

	Number of sessions	Session duration	Strategy rating	Satisfaction with learning	MSR-R score	TSE score	Self-efficacy	Autonomous motivation	Controlled motivation
Session duration	0.042								
Strategy rating	−0.070	−0.022							
Satisfaction with learning	0.014	−0.052	0.698^**^						
MSR-R score	0.060	−0.049	0.091	0.196					
TSE score	0.052	−0.051	0.230^*^	0.358^**^	0.478^**^				
Self-efficacy	−0.108	−0.092	−0.034	0.073	0.211^*^	0.184			
Autonomous motivation	−0.045	−0.251^*^	0.185	0.236^*^	0.419^**^	0.404^**^	0.254^*^		
Controlled motivation	0.068	−0.056	0.120	0.076	0.054	0.019	−0.179	0.131	
Grade	0.111	−0.001	0.164	0.199	0.013	0.212^*^	0.145	−0.020	0.153

* *p* < 0.05

** *p* < 0.01.

**Table 3:** Multiple hierarchical regression: predictors of final course grade.

	*B*	*SE*	Beta	*p*	*R^2^Δ*
**Step 1**					0.025
Constant	60.57	9.90		< 0.001	
Pre-Autonomous Motivation	−0.928	2.34	−0.04	0.693	
Pre-Controlled Motivation	2.91	1.89	0.16	0.126	
**Step 1**					0.038
Constant	48.83	11.49		<0.001	
Pre-Autonomous Motivation	−2.21	2.40	−0.10	0.359	
Pre-Controlled Motivation	3.73	1.91	0.20	0.054	
Pre-Self-Efficacy	0.21	0.11	0.21	0.056	
**Step 3**					0.063
Constant	33.62	14.26		0.021	
Pre-Autonomous Motivation	−3.91	2.61	−0.17	0.137	
Pre-Controlled Motivation	3.85	1.86	0.21	0.042	
Pre-Self-Efficacy	0.20	0.11	0.19	0.070	
Pre-MSR-R	−3.31	3.41	−0.11	0.335	
Pre-TSE	8.54	3.35	0.30	0.012	
**Step 4**					0.011
Constant	33.01	15.02		0.031	
Pre-Autonomous Motivation	−3.75	2.71	−0.16	0.170	
Pre-Controlled Motivation	3.74	1.88	0.20	0.049	
Pre-Self-Efficacy	0.21	0.11	0.20	0.059	
Pre-MSR-R	−3.67	3.45	−0.13	0.291	
Pre-TSE	8.38	3.37	0.29	0.015	
Number of sessions	0.35	0.33	0.11	0.29	
Session duration	−0.001	0.02	−0.01	0.961	
**Step 5**					0.018
Constant	29.95	15.12		0.051	
Pre-Autonomous Motivation	−4.08	2.70	−0.18	0.135	
Pre-Controlled Motivation	3.59	1.87	0.20	0.058	
Pre-Self-Efficacy	0.21	0.11	0.20	0.058	
Pre-MSR-R	−3.66	3.43	−0.13	0.289	
Pre-TSE	7.03	3.49	0.25	0.047	
Number of sessions	0.36	0.33	0.11	0.285	
Session duration	−0.001	0.02	−0.01	0.946	
Satisfaction with learning	2.75	2.01	0.15	0.174	
**Step 6**					0.003
Constant	28.22	15.44		0.071	
Pre-Autonomous Motivation	−4.19	2.72	−0.18	0.127	
Pre-Controlled Motivation	3.51	1.88	0.19	0.065	
Pre-Self-Efficacy	0.21	0.11	0.21	0.052	
Pre-MSR-R	−3.55	3.45	−0.12	0.307	
Pre-TSE	7.02	3.51	0.25	0.049	
Number of sessions	0.38	0.34	0.12	0.257	
Session duration	−0.002	0.02	−0.01	0.921	
Satisfaction with learning	1.64	2.73	0.087	0.549	
Strategy rating	1.41	2.35	0.084	0.551	

#### Motivation, Self-Efficacy, SRL Skills, Study App Usage, and Study App Rating as Predictors of Grades

We performed a multiple hierarchical regression to test the predictors of final course grade. For motivation, self-efficacy, and SRL skills, pretest scores were used. Step 1 consisted of autonomous and controlled motivation, to which self-efficacy was added in step 2. In step 3, MSR-R and TSE scores (measures of SRL skills) were added. Study app duration and usage were added in step 4. Regarding the study app rating variables, average satisfaction with learning was added in step 5 and average strategy rating was added in step 6. This order of entering the variables was based on existing theory supporting motivation (e.g., Vansteenkiste et al., 2009), self-efficacy (e.g., Pajares, 1996), and SRL skills (e.g., Broadbent and Poon, 2015; Dent and Koenka, 2015) as predictors of performance. Accordingly, the novel variables specific to this study (i.e., app usage and ratings) were added in later steps.

As can be seen in Table 3, the results revealed only model 3 (motivation, self-efficacy, and SRL skills variables) to be significant, *F*(5, 96) = 2.61, *p* = 0.030, *R^2^* = 0.125. Within this model, only controlled motivation (*β* = 0.21, *p* = 0.042) and TSE scores (*β* = 0.30, *p* = 0.012) significantly predicted final course grades. Higher controlled motivation and TSE scores led to higher grades on the course exam.

#### Effects of App Gamification Elements

A series of one-way between-subject ANOVAs were performed to compare the two app conditions (gamification vs. standard) regarding students’ app usage, satisfaction ratings of study strategy and learning, and performance (i.e., final grades). Although the Shapiro–Wilk’s test revealed violations of the normality assumption for both conditions on all of these variables (*p* < 0.05) except grades, we carried on with using ANOVA given that it is rather robust to deviations from normality (Field, 2018).

There were no significant differences between participants in the two app conditions on the duration of the sessions, *F*(1, 97) = 0.28, *p* = 0.600, partial η^2^ = 0.003, or the number of sessions *F*(1, 97) = 0.02, *p* = 0.892, partial η^2^ < 0.001. Also, no differences between the two conditions were found for satisfaction ratings for the strategies used, *F*(1, 97) = 0.33, *p* = 0.568, partial η^2^ = 0.003, and for learning, *F*(1, 97) = 0.30, *p* = 0.583, partial η^2^ = 0.003. Not surprisingly, no difference in performance between the two conditions was found, *F*(1, 97) = 2.48, *p* = 0.119, partial η^2^ = 0.025.

In order to compare participants of the two app conditions on changes in motivation, self-efficacy, and SRL skills (i.e., MSR-R and TSE) from the start to the end of the course, two-way mixed ANOVAs were utilized. App condition served as the between-subjects independent variable, while time of measurement (pre-test versus post-test) served as the within-subjects independent variable. The Shapiro–Wilk’s test demonstrated that the assumption of normality was violated for both app conditions on the pre-test measure of self-efficacy, for the standard condition on the post-test measure of self-efficacy, and for the gamified condition on post-test measures of both autonomous and controlled motivation (*p* < 0.05). Nevertheless, we continued with interpreting the ANOVAs given their robustness to non-normality (Field, 2018).

Regarding main effects, the main effect of condition showed a significant difference in autonomous motivation between the gamified and standard conditions, *F*(1, 97) = 5.67, *p* = 0.019, partial η^2^ = 0.055. In particular, participants in the gamified condition had a significantly higher autonomous motivation score (regardless of time point of measurement; *M* = 4.21, *SE* = 0.073) than participants in the standard condition (*M* = 3.96, *SE* = 0.077). Differences in self-efficacy scores between participants in the gamified (*M* = 74.36, *SE* = 1.70) and standard condition (*M* = 69.62, *SE* = 1.79) did not reach significance, *F*(1, 97) = 3.68, *p* = 0.058, partial η^2^ = 0.037. Also, we found no main effects of condition for SRL skills measured by MSR-R, *F*(1, 97) = 0.95, *p* = 0.332, partial η^2^ = 0.010, or TSE, *F*(1, 97) = 2.71, *p* = 0.103, partial η^2^ = 0.027. And no main effect of condition was found for controlled motivation, *F*(1, 97) = 0.97, *p* = 0.326, partial η^2^ = 0.010, and self-efficacy, *F*(1, 97) = 3.68, *p* = 0.058, partial η^2^ = 0.037.

Regarding the main effects of time, there was a significant increase in SRL skills measured by the MSR-R subscale from pre-test (*M* = 3.55) to post-test (*M* = 3.65), *F*(1, 97) = 7.30, *p* = 0.008, partial η^2^ = 0.07. On the other hand, SRL skills measured by the TSE subscale significantly decreased from pre-test (*M* = 4.05) to post-test (*M* = 3.98), *F*(1, 97) = 4.04, *p* = 0.047, partial η^2^ = 0.07. Concerning motivation, there was a significant increase in autonomous motivation from the pre-test (*M* = 4.03) to the post-test (*M* = 4.15), *F*(1, 97) = 6.41, *p* = 0.013, partial η^2^ = 0.06. However, the change in controlled motivation from pre-test (*M* = 2.18) to post-test (*M* = 2.28) did not reach significance, *F*(1, 97) = 3.90, *p* = 0.051, partial η^2^ = 0.04. Lastly, the slight decrease in self-efficacy from pre-test (*M* = 72.20) to post-test (*M* = 72.01) was also not significant, *F*(1, 97) = 0.07, *p* = 0.796, partial η^2^ = 0.001.

The interaction between condition and time of measurement was not significant for SRL skills measured by MSR-R, *F*(1, 97) = 1.36, *p* = 0.246, partial η^2^ = 0.014, or TSE scores, *F*(1, 97) = 0.19, *p* = 0.666, partial η^2^ = 0.002. Also, we did not find an interaction between condition and time for self-efficacy, *F*(1, 97) = 0.65, *p* = 0.422, partial η^2^ = 0.007, autonomous motivation, *F*(1, 97) = 2.03, *p* = 0.158, partial η^2^ = 0.020, or controlled motivation, *F*(1, 97) = 0.02, *p* = 0.967, partial η^2^ < 0.001.

#### User Friendliness

A five-item scale with a seven-point answer scale was used to measure user friendliness. It was found that students (*N* = 98) agreed to a moderate extend with the navigation in the Study app (*M* = 4.62, *SD* = 1.73), and the flexibility in changing the content in the app (*M* = 4.06, *SD* = 1.59). The quality of the app measured as the app being “free from errors”(*M* = 4.94, *SD* = 1.55), “up to date” (*M* = 4.99, *SD* = 1.60), and “free from bias” (*M* = 6.05, *SD* = 1.06) was also agreed to by student to a moderate or high extend.

Furthermore, using a five-point scale, it was measured whether students would recommend the Study app to others. The probability that students would recommend the app the fellow students (*M* = 2.78, *SD* = 1.06) or other professional in education (*M* = 2.67, *SD* = 0.95) was moderate.

### Discussion Study 1a

To conclude, although all participants in this study showed an increase in their self-reported motivation and SRL skills, using the Study app did not seem to play a role in this. The results found in Study 1a did not show a positive relation between Study app use (i.e., frequency of sessions or duration) and pretest SRL, motivation, self-efficacy, or course performance measures and therefore do not confirm our hypotheses (H1.1–H1.5). Yet, there was a negative correlation between autonomous motivation and the durations of sessions. It seems that students who were more autonomously motivated for the practical had shorter sessions in the Study app. Possibly, these students just browsed through the app, got the information they were looking for (e.g., on study strategies), then stopped their session but not their self-study activities outside the app and therefore end up with shorter sessions in the app. In addition, it was found that autonomous motivation was positively related to both SRL measures and self-efficacy which is in line with earlier studies (e.g., Baars and Wijnia, 2018; Girelli et al., 2018; Wijnia and Baars, 2021). Interestingly, the TSE scores were found to be related to satisfaction with learning and the learning strategy as rated in the Study app, as well as with the grade for the practical. This seems to suggest that being able to organize learning sessions in terms of time and study environment, is associated with more satisfaction about the learning process and higher performance. As this is a correlation, there is no evidence for the direction of such a relation. Hence, it could very well be the case that higher performing students were also better able to organize their learning sessions in terms of time and environment. From the hierarchical regression analysis, it follows that only controlled motivation and TSE are significant predictors of performance if autonomous motivation, metacognitive SRL, and self-efficacy are included in the model.

The participants in the gamified Study app and the standard Study app both showed similar changes in MSR-R, TSE, self-efficacy, and autonomous and controlled motivation scores from pre- to post-measures and therefore do not confirm our hypotheses (H2.1–2.5). That is, for all students, autonomous and controlled motivation and metacognitive SRL skills increased over the course of 5 weeks. Yet, all students were found to score lower on the TSE indicating that these SRL skills did not improve. However, participants in the gamified Study app conditions showed higher autonomous motivation during the study compared to the participants in the standard Study app without gamification elements. As there were no significant differences in autonomous motivation at the start of the study, these results seem to indicate that having gamification elements in a mobile application to support SRL can help students to sustain autonomous motivation over time which partially confirms Hypothesis 2.2. Interestingly, sustained autonomous motivation could be beneficial for SRL (Baars and Wijnia, 2018; Girelli et al., 2018; Wijnia and Baars, 2021) and performance (e.g., Vansteenkiste et al., 2009).

There are some limitations to this which should be taken into account when interpreting the results. First, the participants in the sample chose to participate in this study, which might have created a selection bias in our sample. Second, there is a gender imbalance in our sample as we have more women than men. Also, the scales used to measure SRL skills (i.e., MSLQ, MSR, metacognitive self-regulation and TSE, and time and study environment) turned out to have a low reliability, which means they should be interpreted with caution. Furthermore, from the trace data collected in the application, it is unclear how long a participant was actually active during a session which made it difficult to pinpoint duration of sessions. That is, participants could have been doing something else while the application was still running. Future work on this type of applications could look into more detailed ways of measuring engagement with the application to get a better estimate of the session duration. Finally, although we did not have specific hypotheses about the exact number of sessions that participants should have used the study app, from the descriptive statistics in Table 1 it is clear the number of sessions is quite low (i.e., on average four sessions across 5 weeks). Also, the variety in strategies that were chosen is quite low. That leaves us with the open question as to why this number of sessions and the variety of strategies is low. In Study 1b, these issues were investigated by means of focus group interviews that were taken after the practical and Study 1a had ended.

## Study 1b: Students’ Experiences Regarding the Ace Your Self-Study App

Based on the literature, it is clear that people generally overestimate their learning performance (e.g., Dunlosky and Lipko, 2007; Bjork et al., 2013), which could potentially harm subsequent study processes (e.g., Dunlosky and Rawson, 2012). In addition, students are often not aware of effective study strategies (McCabe, 2011; Dirkx et al., 2019) or how to use them effectively (Bjork et al., 2013). Therefore, we assumed that providing support *via* a mobile application offering students guidance and support throughout the different phases of SRL (i.e., forethought, performance, and reflection, Zimmerman, 2008), would benefit their SRL skills, self-efficacy, motivation, and lead to better performance (i.e., course grades) at the end of a 5-week course (Study 1a). However, although students improved their SRL skills and motivation across the course, students’ usage of the Study app was relatively low, the variety of strategies that were chosen during study sessions was low and, the usage of the Study app did not significantly predict performance. This result is possibly related to the fact that students had to decide how to study and whether they would use the app (apart from two assignments in which they were explicitly invited to use the Study app). This required students to be able to accurately reflect on their learning processes and decide whether they would need and how they would use the offered guidance and support.

Nevertheless, the precise reasoning behind students’ self-study decisions was not captured in Study 1a. Therefore, in Study 1b, the experiences of a subset of students participating in Study 1a were investigated. That is, using a qualitative approach, students’ experiences with their self-study activities were investigated retrospectively *via* focus group interviews to gain more insight in student’s experiences and reflections on their self-study sessions in the context of Study 1a.

In order to understand students’ self-study activities and how students used the Study app during self-study sessions in Study 1a, we investigated (a) students’ study behaviors during self-study sessions in general and (b) students’ experiences with the Study app during their self-study sessions. Three main research questions were formulated accordingly:

1. How much time did participants spend studying for the course (in terms of both duration and quantity of study sessions) with the Study app compared to without the Study app, and why?
2. Does participants’ choice of study strategies differ when studying with and without the Study app, and why?
3. What would motivate participants to try new or unfamiliar study strategies?

### Method

We created an OSF page for this project, where all materials and a detailed description of the procedure are provided (DOI 10.17605/OSF.IO/98NYH).

#### Participants

Eleven first-year Psychology bachelor students (eight females, three males) aged between 17 and 36 (*M* = 20.91, SD = 5.54) who had participated in study 1a voluntarily participated in the focus group interviews. Specifically, out of the five invited students per focus group, three students participated in focus group 1, four in focus group 2, two in focus group 3, and two in focus group 4. Which focus group a student participated in depended on the student’s availability regarding date and time. In return for their participation, the students received research credit of 30 min, which contributes to completing mandatory research hours as per the requirements of the bachelor program. Although all participants had been introduced to the self-study app in practical 1.1 (study skills), not everyone tried using the app or made use of it throughout the course as instructed.

#### Design and Instrument

Semi-structured focus group interviews with students who participated in Study 1a took place after the 5-week course during which Study 1a was performed. An interview guide was created and used during the focus group meetings. This guide consisted of a list of questions to be covered during the interviews, divided into topics according to the research questions. Following a deductive approach (Crabtree and Miller, 1999), the interview topics were based on a set of *a priori* themes established from a review of the literature, the research questions and our professional experience in teaching first-year university students. Each section in the interview was allocated a time duration, which was estimated based on the complexity of the questions and a pilot study (i.e., focus group 1). Furthermore, we created slides containing the interview questions and some relevant data obtained from study 1a that helped to support or elaborate on these questions (see Appendix E). For topic 3 on study strategies and topic 4 on motivation to use study strategies, we added Figure 5 showing an overview of the percentage of sessions during which one of the types of learning strategies was used, on the slide to help students reflect on the questions concerning the topic. The slides were printed out on A4 paper to be distributed to the participants and interviewers during the focus groups. Each focus group was audio recorded. Additionally, one or more of the interviewers used a notepad and pen or laptop for note-taking.

#### Procedure

Four focus group sessions were organized in total. Students who had provided their email addresses to be invited for a focus group interview at the end of the first survey in Study 1a were invited to participate in a focus group *via* email. After the fourth focus group session, no further sessions were planned as we noticed saturation in terms of the information provided by the participants. For each focus group, one of the two researchers present had the role of the main interviewer, and the other made an audio recording and notes. The interviewer obtained consent from the participants for recording the conversation and informed the participants that the recordings would be treated confidentially and used for research purposes only. The interviewer went through the questions, giving each participant a chance to answer and respond to each other’s input.

#### Analysis

All focus group audio recordings were transcribed verbatim and then coded, organized, and analyzed. Consistent with a data-driven, inductive approach (Boyatzis, 1998), no predetermined structure was used to analyze the data. This exploratory approach allowed themes to surface directly from the interview data. As some focus groups were quite small (i.e., only two persons), we chose the individual as the unit of our analysis and performed a content analysis. First, two of the researchers carefully read all transcripts, and key topics were identified per transcript (i.e., coding). Then, they summarized the raw data of each participant according to the topics that emerged. Additionally, quotes were extracted per topic. Subsequently, the summaries and quotes across all transcripts were combined according to the recurring topics, from which the following higher-order topics were derived: Self-Study Sessions, Choice of Study Strategies, and Motivators to Use New and Unfamiliar Study Strategies.

### Results

#### Self-Study Sessions

It was quite common for students to not keep track of their study sessions in terms of duration and amount. Nevertheless, some estimations were given, supported by explanations. The average duration of self-study sessions for tutorials (course work) reported by the students had much variation. Some students reported shorter durations, such as 2–3 h, while others reported long durations of up to 8 h. A few students indicated that their study schedule is more tentative than planned; they study when they feel like it and stop studying when they do not feel like studying anymore, or when they have lost focus.

*“If I want to study then I can just focus for a couple of hours straight, and then if I do not feel like studying then I just stop.”*

Some commented that the length of the study session depends on external factors such as how busy a given day or week is and the difficulty level of the learning material. More time is spent studying when the study material is of a higher difficulty level. Not all students mentioned whether they take breaks, but those who did, reported longer study sessions said they take lots of breaks in between, whereas those who reported shorter study sessions reported a short break of around 10 min per session.

When it comes to studying length for app users, students tended to forget about the app during a study session. Hence, they forgot to switch it off; the app kept running long after their actual study session was over. As a result, students estimated the length of their study sessions with the app to be around 2 h when they did use it.

*“One of the things that was very challenging in the app is that I cannot pause it and come back to it.”*

Answers regarding how often the students studied per week varied from studying every day to studying 2–3 times a week (mainly as preparation for the tutorial groups that take place twice a week). Some students reported having a fixed routine, for instance: studying 5 days and having 2 days completely off. Others reported a more variable study routine, depending on what other activities there are to do in a given week and how efficient one’s studying is on a given day. If inefficiency is high, students would instead stop studying and rest. One student mentioned the tendency to do things last minute due to procrastination and not feeling like studying, which results in mass studying the day before the tutorial meeting. Some students adjusted their amount of study sessions over time as they progressed with the course; for instance, one student increased his/her number of study sessions, whereas another student reduced the number of study sessions due to burnout.

The purpose of using the Study app for self-study sessions differed among students; some only utilized it around the exam period, while others used it only for preparing for the practical.

*“Only used the study app around exam period, did not use it that much.”*

Only one student used the app quite often, and the given reason for this was that the app was assigned for class, and the student was self-disciplined. On the other hand, a few students would have liked to use the app more than they did for the following reasons: it may have helped attain higher grades, added structure, and lower procrastination and stress levels.

Most students did not use the study app much; they tried it once or twice. A common reason for this was that most students did not think they needed it to study better. One explanation for the overall low frequency of app usage was that one would only be motivated to use the app if his or her study method is not working, or he or she is getting bad grades.

*“I would’ve liked to use it more, maybe to add more structure, because I also procrastinate and just do it the day before.”*

*“I could try* [using the app] *maybe more because I want higher grades.”*

Another explanation for the low app usage frequency was that the students did not receive an adequate explanation of its purpose and how it works beforehand, so they did not find it very useful. They downloaded it because they were asked to, but they did not end up genuinely understanding it and how it can help to study. Some students would not have used the app more than they did for various reasons. Some explanations were more linked to personal study habits and preferences, such as not spending much time preparing for tutorials, and therefore only needing the app to prepare for the exams.

*“If it’s like you always have to set goals, set time for studying, set study materials, and set when you are supposed to be finished, I find that very restricting and very boring and very stressful.”*

Another reason was that the student already plans everything and works ahead of time, so the app is redundant for this purpose. Other explanations for not using the study app more pertained to the app itself. Some students found the app distracting and not helpful. They mentioned that the app does not take variability across individuals into account; what method is effective varies across people. This app was said to be more suitable for students who are completely lost about their work method.

To summarize, there was quite some variation in the length of study sessions and the number of sessions per week. Concerning using the Study app for self-study sessions, students’ responses seem to show a difference in how the app was perceived across students, and one’s perception about their study situation plays a role herein. In general, those who think they know what they are doing in terms of self-study will think they do not need to use a self-study app.

#### Choice of Study Strategies

Study strategies commonly used by students were summarizing (including altering/revising summaries after tutorial groups), note-taking (e.g., of keywords and definitions), self-explaining (or explaining to someone else), organize and elaborating, and relating concepts and theories (e.g., through a chart). Highlighting relevant parts from the literature, brainstorming, and using flashcards were also mentioned. Generally, the strategies reported were relatively homogeneous across the students. The reason given by the students for using these strategies is that they are familiar and effective.

*“Well, I already sort of do these already, so I just inputted the ones I already [use].”*

*“I always summarize. It’s just easy for me and I’ll have like a whole picture off what’s, how do you say that, yeah, what I need to study.”*

Students reported that, especially under time pressure, one needs to be efficient by sticking to the known-to-work strategy. A noteworthy observation is that sometimes students incorrectly labeled a strategy, such as referring to highlighting as summarizing.

*“That’s considered summarizing, and sometimes I use if I have time or if I’m not lazy, too lazy, I sometimes write it down instead of just highlighting it.”*

Following the strategies students utilized when studying in general, students commonly used summarizing, note-taking, organize and elaborating, and self-explaining in the self-study app (i.e., familiar strategies). Additionally, some students tried concept mapping. This strategy suited a student with visual memory but did not suit another student’s style, who used clear writing goals instead. However, once students learned how to use a strategy provided by the study app, they no longer found it necessary to use the app to continue utilizing that strategy. This indicates reduced benefit of the app once the desired strategy is learned.

Furthermore, students never attempted to use some of the strategies due to a lack of fit to one’s personal preferences (e.g., drawing), while others were perceived to be too specific and not necessarily suited to self-study (e.g., brainstorming). Importantly, students mentioned that not all of the strategies offered in the app were familiar to them, which made them less likely to use the app, especially under time pressure. In such situations (e.g., exam week), students are more likely to use strategies that have worked for them in the past and avoid taking risks with unknown strategies.

*“Uh I have a question because organize and elaborate is little bit same like summarizing right? Or not.”*

*“Um, really it’s because when time becomes when you are under pressure, you are going to just switch to what you know, and what you know will get you the result you are looking for, so everything else gets thrown out, and until I know until I have enough practice with these.”*

Overall, participants’ study strategies did not largely differ when studying with and without the study app. The reason is that students are likely to stick with what is already familiar to them. Students avoid taking risks with new study strategies, especially under time pressure, which is tied to their perception that unfamiliarity decreases studying efficiency, as more effort is required to understand the strategy and utilize it properly. Students’ general unwillingness to learn new, unfamiliar study strategies may have contributed to the low amount of app usage observed in this study. In other words, students may not feel an added value of the app if they decide to stick to known strategies that they perceive to be effective and efficient, especially when there is time pressure (which may often be the case due to a high workload, procrastination tendencies, or both).

#### Motivators to Use New and Unfamiliar Study Strategies

A commonly-mentioned factor that would motivate students to attempt new strategies was low grades. Students would take this as an indicator that one’s current strategy is not working.

*“Bad grades; if my strategy is not working, then I have to change my strategy.”*

More specifically, studying very hard but not getting the grades one desires would indicate a problem with one’s current strategy. There was agreement that low grades lead to consideration of what can be done differently when studying. However, so far, this had not happened for the students we interviewed, and therefore they found no need for learning new strategies.

Another mentioned motivator to try a new strategy would be if the topic of study changes, which requires a different approach.

*“Yeah if the topic changes, so like for statistics it’s a different approach to just reading stuff, so I’m gonna do different things.”*

For example, mathematics is studied in a completely different way than biology. In addition, the absence of time pressure would also motivate students to attempt new strategies. Regarding the strategies provided by the app, students often found them too time-consuming to learn. Taken together with the time pressure that most students felt, the strategies provided in the app were perceived as inefficient. Furthermore, according to the students, a thorough explanation of the strategies provided by the course instructors would have contributed to efficiently learning the study app strategies. Other suggested motivators include discussing study strategies with peers to understand what works for others.

*“no inner drives would motivate me to seek a new strategy, but if someone from outside delivers something that I find very interesting and very unorthodox, I might try using it and if it’s good for me I might do it again.”*

Taken together, the answers given by the students point to external factors as motivators to try new study strategies.

### Discussion

In general, when it comes to self-study experiences and reasons for using the app, it seems that as long as students feel comfortable with their study habits, they are not inclined to seek help or change their strategies. Students tend to avoid the effort or risks they perceive to be involved in changing study strategies. In line with the findings by Biwer et al. (2020), it seems that perceived time and effort play an essential role in making changes in SRL during self-study. Possibly time management could be a prerequisite for students to engage in SRL and using effective study strategies. Furthermore, students’ general unwillingness to start using unfamiliar study strategies might have prevented them from using the Study app to support their self-study sessions to some extent. Overall, participants’ study strategies did not differ much between studying with or without the study app. In line with earlier studies (McCabe, 2011; Bjork et al., 2013; Dirkx et al., 2019), the current study showed that students primarily used strategies that were already familiar to them, did not use other strategies that were offered, and sometimes mislabeled strategies during the focus group interviews. These findings seem to indicate a lack of knowledge and expertise about using study strategies in general. Moreover, from a motivational perspective, it seems students are not very interested (i.e., intrinsically motivated) in how to regulate their learning during self-study and what study strategies are useful nor find it very relevant (i.e., identified motivation). That is, as long as their grades are fine, most students do not seem to give their SRL activities much thought. Some students do indicate that they might be more willing to think about their self-study habits and study strategies if someone would explain the relevance of it. Hence in line with the SDT (Deci and Ryan, 2000; Ryan and Deci, 2000a,b, 2020; Pelletier et al., 2001), it seems promising to explain to students more about the relevance of SRL activities and using effective study strategies to support their motivation for engaging with them. Indeed, several studies have shown that informing students about the relevance of specific aspects of SRL (e.g., training in applied memory and learning topics, McCabe, 2011; informing about making overconfident judgments, Roelle et al., 2017b), can benefit actual SRL.

These results do need to be interpreted with caution. That is, the slides used to present the questions on study strategies and motivation to use study strategies in the focus groups also contained data on the study strategies that were used in Study 1a. This might have influenced students’ responses. Moreover, as a consequence of using semi-structured focus group interviews with preselected topics and questions, our analysis cannot be classified as truly inductive. Furthermore, some of the focus groups were quite small and this might have prevented students from having a discussion about the topics and questions that were presented. Future research could use in-depth interviews as a method to investigate students’ experiences with self-study sessions and SRL support such as the Study app.

## General Discussion

All first-year students in our sample improved their self-reported motivation and SRL skills over the course of 5 weeks, during which they followed a practical on how to study (i.e., Study 1a). Yet, having the Study app to support the phases of SRL and using a variety of study strategies was not related to this increase. Students only used the app for a few sessions and largely stayed with the study strategies they most likely already knew (e.g., note-taking or summarizing). From focus group interviews (i.e., Study 1b) about self-study, study strategy use and using the Study app, it seems students believe the support offered in the form of the Study app was not always what they needed. Results from Study 1b showed that motivators to seek support or try out new study strategies were often external, such as grades students receive.

Based on findings from earlier studies, supporting all three phases of SRL (e.g., Dignath and Büttner, 2008; Wong et al., 2019) and offering guidance on how to use study strategies (e.g., McCabe, 2011; Bjork et al., 2013) was hypothesized to help students employ SRL strategies and thereby improve their SRL, motivation, self-efficacy, and performance across a first-year course. We found SRL skills to be related to autonomous motivation and self-efficacy as expected (Baars and Wijnia, 2018; Girelli et al., 2018; Wijnia and Baars, 2021). However, our findings showed that using the Study app does not affect these relations significantly. In other words, students improved their SRL skills and motivation across the course regardless of their usage of the Study app. Furthermore, we found most students used the app only for a limited number of sessions. For many students, the support offered *via* the Study app was not perceived as fitting to their needs.

As witnessed in Study1b, students have their personal reasons for seeking and using support for SRL and study strategies. Some said the app was useful for preparing for the exams, but others said it was useful for preparing meetings. Moreover, some students indicated the app was not helpful to support their self-study at all. There might be a fundamental issue with the idea of having learners decide how they would like to go about their learning and what support they might need. Although we would like learners to become effective self-regulated learners, they might not be able or equipped to do this. Learners who might need help or support are not always the ones who ask for help or use the support offered (e.g., Ryan et al., 2001; Karabenick, 2003). Our results resonate with these findings and underline that there are several different factors, such as personal motivational characteristics (e.g., Ryan et al., 2001) or the social context of learners (e.g., Won et al., 2021), that play a role in seeking help or using support during self-study. For example, students in our study explained that as long as they were convinced that they did all right (e.g., obtained good grades); they did not see the need to use support during self-study. Possibly, this points toward an “experienced-learning-versus-actual-learning-paradox” in which student are overconfident about the effects of their self-chosen strategies.

As mentioned earlier, some students explained that the app did not suit the individual needs of students. The application might have been too general or not in line with students SRL knowledge or skills to be useful for all. Another possibility would be that the app’s implementation could have been more successful when combined with instruction on what SRL is and why it is important. Findings by Broadbent et al. (2020) have shown that combining a mobile application to monitor learning with online SRL training helped students improve their resource management, cognitive, and metacognitive strategies. Future research could investigate the combination of SRL training with the Study app to improve SRL.

Moreover, from the focus group interviews in Study 1b, it also became clear that after students learned about a strategy *via* the app, they sometimes decided not to use the app any longer as they already mastered a new strategy and were no longer in need of other or more support. These findings suggest that the application was not adaptive to the needs of the students. By using adaptive technologies (e.g., Molenaar et al., 2019; Peng et al., 2019), applications such as the Study app could potentially create a more personalized way of SRL support to ameliorate these issues. Future research could investigate how more adaptive applications can be developed for supporting SRL during self-study.

If students used the Study app, results showed that satisfaction with the chosen strategy and satisfaction with learning during that session in general were significantly correlated. Although this result does not show the direction of the relation, it does underline the importance of study strategies for self-study. In line with other studies (Dignath and Büttner, 2008; McCabe, 2011; Biwer et al., 2020), our results suggest that it is promising to support students’ strategy knowledge and use to improve their learning processes.

As SRL skills were related to motivation (Vansteenkiste et al., 2009; Baars and Wijnia, 2018; Wijnia and Baars, 2021), it is important to take a look at the results of the current study in terms of motivation. Autonomous motivation increased from pre- to post-test but controlled motivation did not. Interestingly, as motivation can be placed on a continuum (Deci and Ryan, 2000), perhaps participants’ motivation moved along the continuum during the 5 weeks of the course. Amongst the students who used the app, half of them got to use a gamified Study app, and the other half got to use the standard Study app. We expected that the gamification elements would keep students more engaged and motivated (*cf.* Su and Cheng, 2015; Mekler et al., 2017) to use the Study app compared to the standard Study app. As motivation increased for all participants regardless of whether they had used the gamified or standard app, this hypothesis was not confirmed. Interestingly, we did find a main effect of condition on autonomous motivation. That is, participants in the gamified Study app conditions showed higher autonomous motivation during the study compared to the participants in the standard Study app without gamification elements. As there were no significant differences in autonomous motivation at the start of the study, these results seem to indicate that having gamification elements in a mobile application to support SRL can help students to sustain autonomous motivation over time. Whether this result can be explained by using the gamified Study app or whether the participants in the gamified condition had slightly more autonomous motivation overall independently from the app seems to be unclear. Future research could look into these possible changes in motivation across weeks at an individual level to gain a better understanding of motivation in relation to SRL. Furthermore, no differences in the use of the Study app, SRL skills or self-efficacy were found. Future research could look into what type of gamification element are attractive to students when using mobile technology for SRL and how it affects motivation, SRL, and self-efficacy.

To conclude, first-year students were offered a mobile-application to support their SRL during self-study sessions across a 5-week course. Moreover, a subset of these students participated in focus group interviews about their experiences with the self-study sessions, the mobile application, and strategy use. Although all participants in this study showed an increase in their self-reported motivation and SRL skills, using the Study app did not seem to play a role in this. Based on the focus group interviews, it seems that students did not always see the need for using the app as support for their self-study sessions. Yet, it was also shown that students mostly used the same, already familiar, study strategies and would only change this if external motivators such as low grades would force them too. Possibly, it is too difficult for students to understand when or why they would benefit from SRL support or how this type of SRL support could help them regulate their learning during self-study sessions more efficiently. Possibly a more firm connection to the curriculum, with an increased involvement of teachers and tutors in the process, or a training for students to understand why SRL is important, could ameliorate these issues. An important takeaway here is that just offering support for SRL in an easy to use and attractive way does not mean that students will use the support and benefit from it.

## Data Availability Statement

The raw data supporting the conclusions of this article will be made available by the authors, without undue reservation.

## Ethics Statement

The studies involving human participants were reviewed and approved by Research Ethics Review Committee of the Department of Psychology, Education & Child Studies. The patients/participants provided their written informed consent to participate in this study.

## Author Contributions

MB, SK, and LR contributed to conception and design of the study, organized the data files, and wrote sections of the manuscript. SK and MB performed the statistical analysis. MB wrote the first draft of the manuscript. All authors contributed to the article and approved the submitted version.

## Funding

This research was funded by the Community for Learning and Innovation (CLI) at the Erasmus University Rotterdam.

## Conflict of Interest

The authors declare that the research was conducted in the absence of any commercial or financial relationships that could be construed as a potential conflict of interest.

## Publisher’s Note

All claims expressed in this article are solely those of the authors and do not necessarily represent those of their affiliated organizations, or those of the publisher, the editors and the reviewers. Any product that may be evaluated in this article, or claim that may be made by its manufacturer, is not guaranteed or endorsed by the publisher.

## Footnotes

^1^Baars, M., Zafar, F., Hrehovcsik, M., de Jongh, E., and Paas, F. (submitted). Ace your self-study: A mobile application to support self-regulated learning. Manuscript submitted for publication.

^2^ www.qualtrics.com
