# Supplementary material for: Exploring Students’ Use of a Mobile Application to Support Their Self-Regulated Learning Processes
Source: Front Psychol. 2022 Mar 14;13:793002. doi: 10.3389/fpsyg.2022.793002 (PMC8964128; doi:10.3389/fpsyg.2022.793002)
Supplement: Supplementary file 1 [file Table_1.DOCX]

**Appendix A: Strategies organized by type of task**

| **Type of task** | **Strategy** | **Description** |
| --- | --- | --- |
| **Studying text** | Organize and elaborate | Make an outline, graphical organizer, flowchart or matrix of the major topics and ideas in the materials you have to study. |
|  | Note taking | Take notes about the main ideas and supporting details from the text in a notebook to keep paying attention to the materials you are studying. |
|  | Summarizing | Make a summary of the most important topics in the text. |
|  | Concept mapping | Make a concept map in which the most important concepts from the text placed in boxes which are connected to each other using lines to show the relation between concepts. |
|  | Mnemonics (memory tricks) | Create memory tricks to remember the materials you are studying. For example, find associated words, associated images, a rhyme or story which relates to the materials to help you remember. |
|  | Self-testing | Give yourself a practice test, to test your own understanding. |
|  | Self-Explaining | Explain in your own words the main ideas from the text. |
|  | Drawing | Make a drawing that depicts the text. |
|  | Imagining | Imagine a drawing that depicts the text. |
|  | Spacing | Spread repetitions of the learning content over time. Instead of studying long and intensively but only once, repeat learning content. For example repeat content from last week or the week before. |
|  |  |  |
| **Problem solving assignments (e.g. math, economics, statistics)** | Generate-and-test | If you have a problem or assignment with a limited number of solutions, generate the possible solutions and test them. If the first solution does not work, move on to the next most likely solution to the problem and so on. |
|  | Analogical reasoning | Find an analogy between the problem or assignment you are working on and a familiar situation. This familiar situation can help you to find the solution to the problem you are working on.  Make sure the analogy is really similar in its structure to the problem or assignment you are working on. Only the details should differ. |
|  | Brainstorming | First, define the problem or assignment you are working on. Second, formulate possible solutions. Third, decide on criteria for judging the solutions you have generated. Fourth, use these criteria to select the best solution. Do not be too critical in the second step, keep room for creativity. |
|  | Worked-out examples | Find a worked-out example in which the solution path (steps) and the solution to the problem or assignment you are working on is shown. You can study the worked-out example to learn how to solve the problem or assignment you are working on. |
|  | Self-testing | Use a similar problem solving task to test your own understanding. |
|  | Self-Explaining | Explain in your own words how to solve the problem you are working on. |
|  | Drawing | Make a drawing that depicts the problem or the assignment. |
|  | Imagining | Imagine a drawing that depicts the problem or the assignment. |
|  |  |  |
| **Writing assignments** | Organize ideas for writing | Before you start writing, locate the information you need, generate writing ides, and organize them. You can use an outline, graphical organizer, flowchart or matrix to do so. |
|  | Models for writing | Find a model text of the type of text you are planning to write. Carefully analyze the critical elements in order to be able to use these elements or techniques from the model text in your own text. Note, use it as an example, do not plagiarize! |
|  | Clear writing goals | State specific goals about the written product you are about to write or about the revision you are about to make to a written product. Specify what you are planning to write or how you are planning to revise. |
|  | Plan-draft-revise | Work in cycles of planning what you want to write, making a draft of that and revise your writing. Repeat these cycles a couple of times. Note, you can do this together with other students. |
|  |  |  |
| **Test anxiety** | Write about your worries | Write down your exam related thought right before your exam. Writing may elevate the burden of your worries about your exam on your memory and help you perform better. |
|  |  |  |
| **Other** | *All of the above strategies can be chosen* |  |

**Appendix B:** **Challenges**

| Name | Description |
| --- | --- |
| \| Lucky number \| \| --- \| \| Crazy number \| \| Crime number \| \| Making it stick \| \| Well-read \| \| Librarian \| \| For research purposes \| \| Resolution for everything \| \| What are boundaries \| \| The hungry catapillar \| \| Isn't it poetic? \| \| Shakespeare \| \| Tiem trial \| \| Streak \| \| Streak \| \| Streak \| \| That's nice honey \| \| Blurred lines \| \| Michelangelo \| \| Rock star \| \| Know-it-all \| \| Are you Pondering what I'm Pondering \| | \| Use 7 different strategies \| \| --- \| \| Use 11 different strategies \| \| Use 17 different strategies \| \| Use 3 different strategies in Studying Text \| \| Use 7 different strategies in Studying Text \| \| Use 10 different strategies in Studying Text \| \| Use 2 different strategies in Problem solving \| \| Use 4 different strategies in Problem solving \| \| Use 7 different strategies in Problem solving \| \| Use 2 different strategies in Writing assignments \| \| Use 3 different strategies in Writing assignments \| \| Use 4 different strategies in Writing assignments \| \| Finish a 1-hour session \| \| Finish at least one session 3 days in a row \| \| Finish at least one session 5 days in a row \| \| Finish at least one session 7 days in a row \| \| Use the Drawing strategy 1 times \| \| Use the Drawing strategy 3 times \| \| Use the Drawing strategy 7 times \| \| Get 3 stars on 3 different strategies \| \| Have a total study time of 50 hours \| \| Finish a Brainstorming session with another person 3 times \| |

**Appendix C: Homework and reflection assignments**

*Downloading and installing the app*

**How to download and install the app (10 min)**

1. Go to the App or Play store or <https://aceyourselfstudy.nl/> or use the QR code.


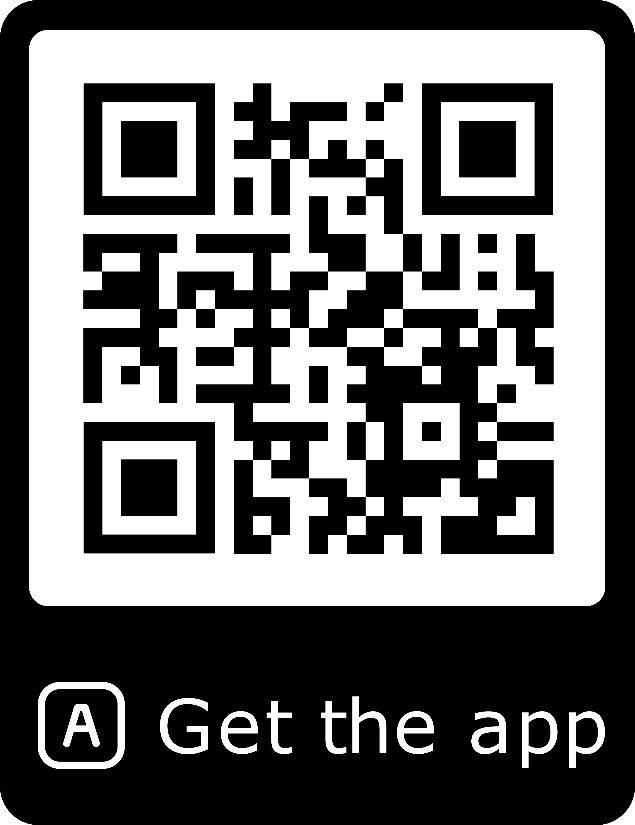


2. Download the Ace your self study app

3. Register using **your student account (!).**

*Homework*

Use the Ace your self study app for your self-study sessions for the course during the next weeks. For example, if you have to read a chapter as preparation for the tutorial meeting in the course, use the app.

1. Use the app for **every self-study session** to get an overview of the frequency and duration of your self-study activities.
2. Use **three different strategies** for you self-study activities. This way you can compare the use of the different strategies.
3. Use at least 2 strategies you **have not used before** to study.

*Reflection assignment*

Interactive session on self-study activities based on Ace your self study app

1. Discuss with whole group how much **time was spend on studying and how satisfied** you were in the following two ways:
   1. Stand up and **form a line** based on the **total** study time from the app (Ace your self study app, Log). The tutor will interview random people from the line on how many hours were spend and how that came about.
   2. Form a line again but this time based on the **average satisfaction rating** from the app (Ace your self study app, Log).
2. Make small groups (3 or 4 persons)
   1. Discuss the pros and cons of the strategies in relation to the tasks you have used it for. Try to discuss all the strategies that were used in your group.
   2. Write the pros and cons on a sheet (magic chart) and stick it to the wall or a table.
3. Reflect and come up with improvements
   1. Throw a ball to a random member of the group. The one who gets the ball, provides a reflection (tip **and** top). Go on until everybody got the ball at least once.

**Appendix D: User friendliness**

*Items on navigation and content:*

“The app is simple to navigate.”

“The app offers flexibility to alter content and settings to meet student needs.”

“The content of the app is free of errors.”

“The app feels up-to-date.”

“The app is free from bias (e.g., race, gender, etc.).”

*Scale:* 7-point scale consisting of “Strongly disagree”; “Disagree”; “Somewhat disagree”; “Neither agree nor disagree”; “Somewhat agree”; “Agree”; “Strongly agree”.

*Items on recommending:*

“Would you recommend this app to professionals working in education (e.g., professors, tutors etc.?”

“Would you recommend this app to fellow students?”

*Scale:* “Definitely not”; “Probably not”; “Might or might not”; “Probably yes”; “Definitely yes”.

**Appendix E: Interview guide**

*Focus group interview guide*

1. **Procedure**

**5 min –**

- Welcome and thank you. **Is everyone ok with this conversation being recorded for research purposes?**
- During this interview, we will show some slides with data about the app use during course 1. This is data of a large group of students. We would like to know ***your*** experiences when using the app and ***your ideas and thoughts*** about this.
- The interview will take approximately 30 minutes and is recorded. These recordings will be treated confidentially.
- Please feel free to add and mention more that comes to mind. All your answers are valid and interesting to us. There is no judgment or grading involved in this group interview. We are curious about your experiences and opinions.

1. **Time per session/ number of sessions**

***3 min-***

How long were your self-study sessions on average in course 1.1 (both practical and course/block)? [estimation is fine]

How long were study sessions using the study app compared to study sessions without it? [Estimation is fine]

***3 min –***

How often did you study per week in block 1?

How often did you use the study app for a session? Follow-up question: Would you have liked to use the study-app more or less than you did? And why?

1. **Type of strategies used**

**7 min -**

What strategies did you use when studying in general? And why? [if necessary, please provide an example]

What strategies did you use when using the app? And why?

How familiar were the strategies in the app? And why?

1. **Motivation and SRL questions**

**7 min -**

What would motivates you to start using new or unfamiliar strategies?

What barriers would prevent you from using new or unfamiliar strategies?

1. **Wrap-up / debriefing**

**5 min -**

Is there something that you would like to mention about studying in general of studying with the Study-app that has not been discussed?

- Closing: thank you; opportunity to email Study leader with questions about this research and the outcomes
- Reminder of registering for research hours
